# Racism: the shameful practices that the medical profession is finally addressing

**DOI:** 10.1186/s40695-021-00068-1

**Published:** 2021-11-02

**Authors:** Sherri-Ann M. Burnett-Bowie, Gloria A. Bachmann

**Affiliations:** 1 Endocrine Division, Department of Medicine, Massachusetts General Hospital and Harvard Medical School, 50 Blossom Street, Thier 1051, Boston, MA 02114-2696 USA; 2 Women’s Health Institute, Rutgers Robert Wood Johnson Medical School, New Brunswick, NJ USA

**Keywords:** Discrimination, Racism, Bias, Interpersonal racism, Structural racism, Social determinants of health, Incarceration, SWAN study, WHI, Asian, African American, Black, Indigenous, Hispanic, Latinx, BIPOC

Black Americans represent 13.4% of the United States (US) population but only 3.6% of US medical school faculty [1, 2]. This inequity is due to different manifestations of structural racism, which include disparities in early education opportunities; high cost of undergraduate and medical education; income inequities that make it challenging to pursue medical school or remain in academia; and bias that is experienced by students, trainees, and faculty [3–6]. Disturbing inequities also exist in life expectancies in the US. Even though population level differences in life expectancy between Black and White Americans have been decreasing, certain regions have experienced increasing differences in life expectancy [7]. Specifically, in the last two decades the difference in life expectancy between Black and White Americans living in Washington, DC has increased. In 2016, non-Hispanic Black men residing in Washington, DC lived 17.2 years less than non-Hispanic White men, and non-Hispanic Black women lived 12.1 years less than non-Hispanic White women [7]. Notably, these stark racial differences in life expectancy were predominantly due to differences in heart disease, cancer, and homicide (the latter being for men only with the differences decreasing over time) [7]. Increased tobacco use, decreased physical activity, and obesity contribute to heart disease and cancer and are manifestations of structural racism. Tobacco companies advertise more in communities of color, and many communities of color are unable to engage in regular physical activity because of limited recreational time, lack of green space, or unsafe neighborhoods [8–11]. This special thematic series of Women’s Midlife Health, *Structural Racism and Midlife Health*, focuses on structural racism and midlife health. This topic is particularly relevant now given the ongoing focus on social justice and the growing appreciation of the impact of racism in both society and healthcare. This series includes a review of the Study of Women’s Health Across the Nation (SWAN) by *Lewis* et al, which describes disparities in midlife health in Black and White women; a review of the guidelines from the Women’s Health Initiative (WHI) on the use of race/ethnicity data by *Garcia* et al, which provides a framework on the importance of designing studies that are inclusive of all populations, that use appropriate terminology, and that collect the data that race/ethnicity may be a proxy for, e.g. social determinants of health variables; a review by *Diop* et al that provides important frameworks and language on discussing discrimination with patients [12]; a review by *Hutchinson-Colas* et al discussing how incarceration impacts women’s health; and an interview with Dr. Vivian Pinn, the first director of the Office of Research of Women’s Health at the National Institutes of Health.

Structural racism refers to “processes of racism that are embedded in laws, policies, and practices of society and its institutions that provide advantages to racial groups deemed as superior, while differentially oppressing, disadvantaging, or otherwise neglecting racial groups viewed as inferior” [13]. Structural racism reflects the multiple and reinforcing ways in which societies facilitate racial discrimination through many systems, including housing, education, employment, healthcare, and criminal system practices [3]. These practices lead to discriminatory beliefs, behaviors, and resource allocation; specifically, access to goods, services, and opportunities that are influenced by an individual’s race [3]. Furthermore, structural racism reflects the codifying of these inequities into organizational practice and policy, such that they become the norm. While there has been appropriate focus on discriminatory behaviors that are mediated at the individual level (personally-mediated racism), there needs to be similar focus on structural or institutional racism, as well as internalized racism, which occurs when individuals accept negative beliefs about their abilities and value [14]. Given that structural or institutional racism results in inequitable distribution of resources and opportunities and most of health is determined by social determinants [15], it is imperative that healthcare providers are part of the coalition addressing structural racism.

In education, research, and clinical practice domains, we should be considering how racism, in any of its forms, is at play [16]. When educating future nurses, physicians, and other advanced care providers, we should understand that clinical images must be representative of the diverse populations whom we serve and that race should not be conflated with ancestry [17–19]. Furthermore, we should understand that race is not a risk factor for disease but a proxy for exposure to racism. Thus, our clinical algorithms which include race and in so doing potentially harm vulnerable populations by reducing our ability to diagnose disease should be re-evaluated [20–22]. Similarly, we should be educating about and creating systems to address provider held unconscious bias, which the National Academy of Medicine identified as one of the three root causes of racial and ethnic health disparities, limited access to care and lack of trust in the healthcare system being the other two [23]. Finally, given the deleterious impact of racism or discrimination on health, we should be creating safe spaces for patients to discuss their experiences using a trauma-informed lens. The diversity of research study populations is paramount to ensuring the generalizability of findings [24]. Moreover, investigation also has the potential to advance anti-racism by highlighting and studying the factors that lead to differences in health outcomes (e.g., socioeconomic status or education) versus adjusting for those factors. By placing the spotlight on the connections between social determinants of health or racism and health outcomes, clinical and translational investigators can transform how we understand health and disease considerably [25, 26]. To that end, as the harmful effect of discrimination on health, as measured by allostatic load [27–33], is increasingly recognized, all investigators should consider using metrics like the Everyday Discrimination Scale, as it provides a mechanism by which to examine racism [34–36].

The influence of social determinants of health and/or racism on health and wellness can be invisible because of the pervasiveness and longstanding nature of inequity [37–39]. Social determinants of health are the conditions in which individuals are born, live, learn, work, and play, and their associated health impacts [40–42]. Differences in social determinants are linked to wealth status and drive the powerful association between a person’s zip code and their life expectancy [16, 43]. These conditions, operating across the socioecological spectrum of human life, are influenced by socioeconomic status and by levels of racism. Specific examples of influencers include housing conditions, school quality, environmental conditions, employment opportunities, access to healthy food, and access to quality healthcare, all of which may be influenced by racial inequities [44]. Social determinants of health moderate the downstream biological processes responsible for health outcomes. The disproportionate impact of COVID-19 disease on Asian, Black, Indigenous, and Latinx people reflects longstanding differences in the social determinants of health and the effects of structural racism [45–55]. Health equity (which is the customized distribution of resources and opportunities across a population to ensure no subset of groups is at a particular advantage), not equality (which is the distribution of the same resources and opportunities to every individual across a population), must be our goal [56, 57]. By way of example, let’s consider our approach to two individuals, one who is chronically malnourished with very low body mass index (BMI) and one who has normal BMI. If we give both individuals the same 2000 cal/day diet for 1 week (equality), the chronically malnourished person may improve somewhat but will still languish relative to the normal BMI person. However, if the chronically malnourished individual is given customized meals, with potentially more calories or protein, which differ from the normal BMI person’s nutrition (equity), they will both be positioned to achieve health. Similarly, health equity which addresses the longstanding inequity that has been perpetuated through structural racism and manifests as social determinants of health, should be the focus of our health systems [58, 59].

To progress towards health equity, members of health systems must understand our roles in combatting structural racism [3, 60–64]. To date, the medical profession has not fully acknowledged the history of inequality or bias in medicine and science, which includes the adverse impact of medical racism on BIPOC (Black, Indigenous and People of Color) patients; the continuing negative impact on medical professionals, including BIPOC trainees and staff; and the negative implications on healthcare’s missions of patient care, education, and research [4, 6, 23, 37, 65–73]. Medicine also must publicly acknowledge the unethical research that has been carried out on BIPOC individuals, such as the research-driven gynecological surgeries that J Marion Sims performed on enslaved Black women, including Anarcha Westcott, without their consent or proper anesthesia [74, 75]. Structural racism is embedded and reinforced in medicine’s policies, practices, cultural representations, and norms. Importantly, examples of structural or interpersonal racism, including microaggressions, in science and medicine are increasingly being discussed, which is essential to addressing the invisibility of the problem [76–81].

Currently there is considerable momentum towards addressing structural racism. The diversity, equity, and inclusion (DEI) initiatives that are in development in all major sectors of society (including economic, educational, and health) are pathways to meaningful progress in both eliminating structural and interpersonal racism and promoting health and wellness for all individuals [16, 37, 46, 48, 82]. Despite any associated discomfort, every individual in healthcare delivery, research, education, and administration must feel responsible for directly responding to identified situations of structural or interpersonal racism, challenge currently accepted norms, and advocate for changed policies and procedures. Change will require that we re-evaluate all institutional, state, and federal policies and procedures from an anti-racism/equity lens. This includes how we care for patients, how we educate, and how we mentor those early in career. Additionally, we must appreciate that opportunities are not equally available to all and that race and gender unfairly influence too many outcomes, whether that be a limb preserving procedure, a potentially lifesaving test, or a professional opportunity [83–87]. Knowledge about these inequities is fundamental, in addition there must be authentic individual and institutional commitment to change. We all need shared frameworks to discuss these painful realities, as well as training on how to recognize and respond to both structural and interpersonal racism. Finally, we all need to feel empowered to catalyze the change that is needed to achieve excellence through equity.

## Data Availability

Not applicable.

## Abbreviations

SWAN: Study of Women’s Health Across the Nation
WHI: Women’s Health Initiative
US: United States
